# Epidemiology of Sarcopenia: Determinants Throughout the Lifecourse

**DOI:** 10.1007/s00223-017-0277-0

**Published:** 2017-04-18

**Authors:** S. C. Shaw, E. M. Dennison, C. Cooper

**Affiliations:** 1 MRC Lifecourse Epidemiology Unit, Southampton General Hospital, University of Southampton, Southampton, SO16 6YD UK; 2 National Institute for Health Research Biomedical Research Centre, University of Southampton and University Hospital Southampton NHS Foundation Trust, Southampton, SO16 6YD UK; 3 National Institute for Health Research Musculoskeletal Biomedical Research Unit, University of Oxford, Oxford, OX3 7LE UK

**Keywords:** Sarcopenia, Muscle mass, Muscle strength, Physical functioning, Epidemiology, Risk factors

## Abstract

Sarcopenia is an age-related syndrome characterised by progressive and generalised loss of skeletal muscle mass and strength; it is a major contributor to the risk of physical frailty, functional impairment in older people, poor health-related quality of life and premature death. Many different definitions have been used to describe sarcopenia and have resulted in varying estimates of prevalence of the condition. The most recent attempts of definitions have tried to integrate information on muscle mass, strength and physical function and provide a definition that is useful in both research and clinical settings. This review focuses on the epidemiology of the three distinct physiological components of sarcopenia, and highlights the similarities and differences between their patterns of variation with age, gender, geography and time and the individual risk factors that cluster selectively with muscle mass, strength and physical function. Methods used to measure muscle mass, strength and physical functioning and how differences in these approaches can contribute to the varying prevalence rates will also be described. The evidence for this review was gathered by undertaking a systematic search of the literature. The descriptive characteristics of muscle mass, strength and function described in this review point to the urgent need for a consensual definition of sarcopenia incorporating these parameters.

## Introduction

Sarcopenia is an age-related syndrome characterised by progressive and generalised loss of skeletal muscle mass and strength; it is a major contributor to the risk of physical frailty, functional impairment in older people, poor health-related quality of life and premature death [1, 2]. The condition has recently been recognised as a specific disease by assignment of a single code within the International Classification of Disease [3]. It is responsible for considerable healthcare expenditure, with direct medical costs attributable to the disorder estimated at US $18.5 billion in the United Stated in 2000 [1].

Prevalence estimates for sarcopenia vary widely in different clinical settings, reflecting divergence in the approaches used for definition. Thus, rates of between 1 and 29% have been reported in community-dwelling populations and of 14–33% in residents requiring long-term care [2]. Approaches to definition generally incorporate consideration of muscle mass, strength and physical function. Initial attempts at definition focused around the choice between one of these three measures, for example, measurement of skeletal mass using DXA and estimating the appendicular fat-free mass of the upper and lower limbs, corrected for height or body mass index. Thresholds could be assigned, for example, >2 SD below the sex-specific mean, at which sarcopenia was assigned. It rapidly became clear, however, that the three individual features clustered variably in individuals and differed in the extent to which they predicted harder clinical endpoints such as the risk of falls, fracture, hospitalisation and death. As a consequence, more recent attempts at definition have attempted to integrate information on muscle mass, strength and physical function (Table 1) [4–8].

**Table 1:** Criteria used to define sarcopenia

Study group	Criteria			
	Muscle mass	Muscle strength		Physical performance
ESPEN special interest groups [4]	Percentage of muscle mass >2 SDs below mean in individuals aged 18–39 years in the NHANES III cohort	X		Walking speed <0.8 m/s in the 4-min test or reduced performance in any functional test used for the comprehensive geriatric assessment
European working group on sarcopenia in older people [5]	ALM/h^2^ Men ≤7.23 kg/m^2^ Women ≤5.67 kg/m^2^	Grip strength Men <30 kg Women <20 kg	OR	Gait speed <0.8 m/s
International working group on sarcopenia [6]	ALM Men ≤7.23 kg/m^2^ Women ≤5.67 kg/m^2^	X		Gait speed ≤1 m/s
Society of sarcopenia, cachexia and wasting disorders [7]	ALM/h^2^ of >2 SDs below the mean of healthy persons aged between 20 and 30 years of the same ethnic group	X		Gait speed ≤1 m/s or walking distance <400 m during a 6-min walk
Foundation of NIH sarcopenia project [8]	ALM_BMI_ Men <0.789 Women <0.512	Grip strength Men <26 kg Women <16 kg		X

Since 2010, three such definitional approaches have been developed. The European Working Group on Sarcopenia in Older People (EWGSOP) [5] definition utilised an algorithm with sex-specific thresholds for muscle mass (ALM) corrected for height squared, coupled with grip strength (<30 kg in men and <20 kg in women) and gait speed <0.8 m/s. The algorithm first considered gait speed, and incorporated muscle mass and grip strength in a hierarchical manner. Primary sarcopenia was defined as loss of muscle mass and function deviating negatively from normal ageing without other obvious causal factors. Secondary sarcopenia was designated as loss of mass and function when causal factors other than ageing, such as systemic disease (inflammatory, malignant or endocrine) appeared to be involved.

Soon after, an International Working Group on Sarcopenia [6] developed an algorithm which considered gait speed (<1 m/s) and then incorporated sex-specific threshold values for muscle mass. Finally, the Foundation of NIH (FNIH) Sarcopenia Project [8], applying a classification and regression tree (CART) analytical approach to data from eight predominantly US cohorts, identified thresholds for ALM and grip strength and defined sarcopenia on the basis of weakness with low lean mass, and reduced gait speed with low lean mass. It is now clear that these three definitional approaches yield very different prevalence estimates in the general population of older people. Thus, sarcopenia is found in around 5.5 and 13.3% of elderly men and women, respectively, using the EWGSOP definition, but only in 1.3 and 2.3% of the same sample using the FNIH definition [8].

Several reviews have been published in recent years addressing the epidemiology of sarcopenia using combinations of these definitions. However, we are unaware of any review that focuses on the epidemiology of the distinct physiologic components of sarcopenia, and highlights the similarities and differences between their patterns of variation with age, gender, geography and time or contrasts the individual risk factors that cluster selectively with muscle mass, strength and physical function. To evaluate this issue, we undertook a systematic search of the literature (search terms: epidemiology, muscle mass, muscle strength, physical function) and included studies published up to November 2016. We report here the available measurement methods for muscle mass, strength and physical function, as well as the influence on each of these measures of age, gender, ethnicity, time and other lifestyle and health-related risk factors. Differences in the descriptive epidemiology of these interlinked muscle characteristics will inform novel predictive tools for harder clinical outcomes; shed light on the most effective preventive strategies against sarcopenia and assist the development of more effective clinical definitions for practical and regulatory purposes.

## Methods to Measure Mass, Strength and Physical Function

In order to use muscle mass, strength and physical function to create a universal definition for sarcopenia, appropriate measurement methods must be identified. These methods are often not uniformly suitable for both research and clinical practices. Table 2 shows the range of methods that are used to measure muscle mass and strength. The best characterised and most widely used measure of muscle mass is fat-free mass derived from a whole body dual-energy X-ray absorptiometry (DXA) scan. To obtain a complete picture of body composition, a four-component model comprising total body water, protein, mineral and fat mass is required; however, this is a highly intensive and costly procedure [9]. DXA is ample to produce a three compartment model in which protein and mineral are combined, and distinguished from fat and water. However, DXA is unable to evaluate intramuscular fat, which can account for 5–15% of observed muscle mass in obese people [9]. Isotope dilution, in vivo neutron activation analysis, underwater weight and urinary metabolite estimation are all unsuitable for assessment of muscle mass. Anthropometric methods (arm muscle cross-sectional area, calf circumference and skinfold thickness) are simple but lack precision and are prone to overestimation [10]. Computed tomography (CT) and magnetic resonance imaging (MRI) have high accuracy and repeatability but are limited in their use outside research settings [9].

**Table 2:** Advantages and disadvantages of methods that can be used to measure muscle mass and strength. The methods that are commonly used in research and clinical settings are shown in italics [9, 10]

Measurement methods	Advantages	Disadvantages
Muscle mass		
*DXA*	Three component model combining protein and minerals into “solids”	Unable to evaluated intramuscular fat
*Anthropometry*	Simple to measure	Lack precision and prone to overestimation Inter-observer variation may occur
Urine metabolites	Provides a useful approximation of muscle mass	Unsuitable for research and clinical practice
Isotope dilution methods	Administration of tracers and collection of samples is simple	Unsuitable for research and clinical practice
*Bio*-*electrical impedance*	Easy to use in both research and clinical settings	Lack of standardised methodology May be considered more as a surrogate muscle mass measure than a direct measurement
Air-displacement plethysmography	Highly reproducible	Relies on an assumption that the density of fat mass and fat-free mass are the same in all patients
MRI and CT	More sensitive to small changes than DXA	Large amount of radiation involved
Muscle strength		
Isometric/isokinetic	Recognised gold standard for measuring muscle strength	Cost and availability of equipment
Grip strength	Simple to measure	Variation in methodology makes comparisons between studies difficult Use of standard Jamar dynamometer may be difficult for some patients, e.g. Advanced arthritis

Isokinetic dynamometry is the recognised gold standard for the measurement of muscle strength, but its use is limited by the cost and availability of expensive equipment [9]. Low handgrip strength has consistently been linked with poor health outcomes (long-term disability onset, increased risk of complications, extended hospitalisation) [11, 12]. The first systematic review of objectively measured muscle strength to include a meta-analysis reported a reduction in mortality risk for every 1 kg increase in grip strength across 13 studies involving 44,638 participants [13]. The recommended procedure for measuring grip strength is to take the highest recording out of three repeated tests in the left, and three in the right hand [14]. The Jamar dynamometer is the reference standard for measurement of grip strength; the Martin vigorimeter may be a suitable alternative [14]. Testing for 1 repetition maximal strength (1-RM) using generic resistance exercise equipment is also used to assess muscle strength, but as with conventional quadriceps dynamometry, the equipment is limited to research settings (Table 2).

Physical performance is often measured using the Short Physical Performance Battery (SPPB), an objective assessment tool for evaluating lower extremity functioning in older persons. The SPPB combines the results of balance, gait speed and chair stand tests to give an overall physical performance score [15]. The SPPB has been used to monitor function in older adults as well as having been used to predict risk of certain negative ageing outcomes such as nursing home admission, disability and mortality [11].

## Descriptive Epidemiology

### Age and Sex

#### Muscle Mass

The age-related decline of muscle mass and its negative impact on health were first documented almost three decades ago by Irwin Rosenberg [17, 18]. Subsequent epidemiological studies have described these associations in greater detail [19–22]. Janssen et al. [20] observed reductions in muscle mass appearing in the third decade of life but found it was not until the fifth decade that there were notable decreases in muscle mass. The third decade has also been recognised as a turning point for muscle mass by Silva et al. [21] who identified the age of 27 years as the threshold beyond which skeletal mass begins to be negatively associated with age among both men and women.

Cross-sectional data from men and women in Rochester, Minnesota, showed that men had significantly higher lean body mass and higher skeletal muscle mass than women (56.9 ± 7.8 kg vs 37.7 ± 5.4 kg; *p* < 0.001) [22]. Similar results were found using whole body magnetic resonance imaging in a second sample of 468 US men and women, confirming that men had significantly higher skeletal muscle mass than women (*p* < 0.001) [20].

A number of studies have documented the rate of decline in muscle mass among older adults. Visser et al. [23] reported a change of −0.8% in appendicular skeletal mass in men over a 2-year period and no significant change in appendicular skeletal muscle mass (ASM) in women over the same period. Similar results were observed by Auyeung et al. [24] with a loss of ASM of −1.59% in men and −2.02% in women over a 4-year period. Both studies assessed changes over relatively short periods, and may not have had sufficient statistical power to estimate the precise annualised change in muscle mass with increasing age.

#### Muscle Strength

**Fig. 1:**
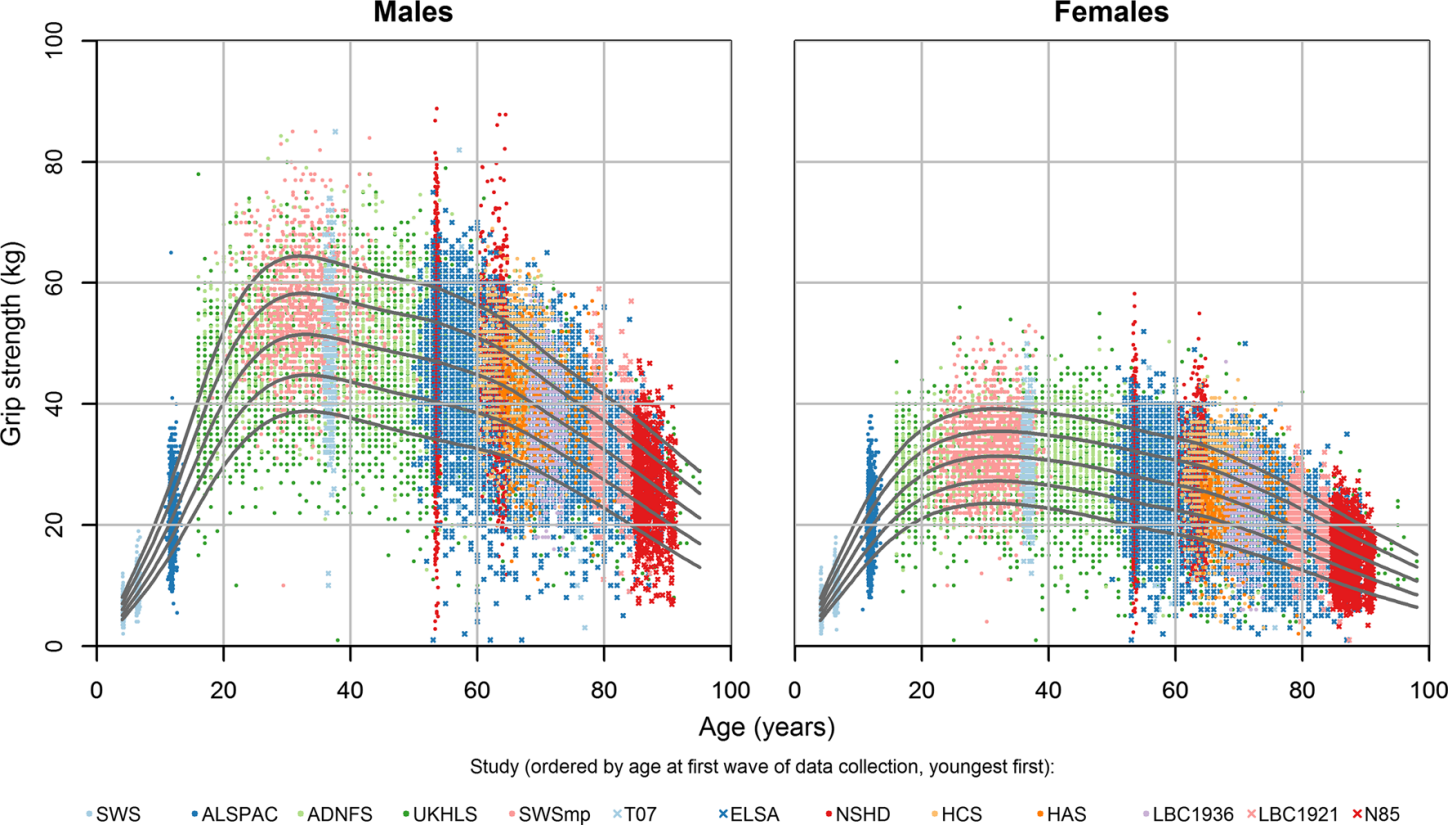
Cross-cohort centile curves for grip strength. Centiles shown 10th, 25th, 50th, 75th and 90th. ADNFS Allied Dunbar National Fitness Survey, ALSPAC Avon Longitudinal Study of Parents and Children, ELSA English Longitudinal Study of Ageing, HAS Hertfordshire Ageing Study, HCS Hertfordshire Cohort Study, LBC1921 and LBC1936 Lothian Birth Cohorts of 1921 and 1936, N85 Newcastle 85 + Study, NSHD Medical Research Council National Survey of Health and Development, SWS Southampton Women’s Survey, SWSmp mothers and their partners from the SWS, T-07 West of Scotland Twenty-07 Study, UKHLS Understanding Society: the UK Household Panel Study [25]

A pooled analysis of data from several UK cohort studies has recently produced a cross-sectional centile curve (Fig. 1) of grip strength across the life course; the study suggested three overall phases of change in muscle strength: an increase to peak in early adult life, maintenance through to mid-life and decline from mid-life onwards [25]. It has been well documented that the age-associated loss of strength is more pronounced with advancing age [19, 26–28]. Comparisons between men, in the most extreme age groupings (20–29 years, 85 + years) included in the InCHIANTI study, showed that knee-extension torque and hand grip strength were approximately 50% lower in the oldest age group (*p* < 0.001) [28].

A number of studies have reported on sex differences in muscle strength with men on average having higher strength than women [10, 16–20], a difference shown to be evident from adolescence onwards [25].

The rate of decline in strength with age appears to be much greater for both sexes than those of muscle mass. The Health, Aging and Body Composition Study (Health ABC) showed that the annual rate of leg strength decline was 3.6% in men and 2.8% in women after accounting for the greater initial strength of men at baseline [19]. Similar results have been shown in other populations [29, 30] and suggest that participants who have greater muscle strength at baseline tend to experience faster rates of strength decline than those who are weaker at baseline. Selective mortality of those in the weakest baseline category and regression to the mean are also potential contributors to this pattern.

#### Physical Function

Data from the Healthy Ageing Across the Life Course collaboration (HALCyon) revealed greater physical capability among younger participants than older participants (*p* < 0.01 for trend across 5 year age groups), in the majority of tests of physical function [29]. Men also performed better in most of the physical capability tests but this gender difference was attenuated for gait speed after adjustment for body size [29].

Physical function in healthy older adults aged 68–82 years, declined on average by 11% in women and 9.6% in men over a 3-year period in The Québec Longitudinal Study on Nutrition and Successful Aging (NuAge) [31]. Similarly, Cooper et al. [29] observed a divergence in the difference between men and women with advancing age for walking speed, with women experiencing a faster rate of decline than men. As with muscle strength, Peeters et al. [32] present evidence from three female cohort studies to suggest that greater rates of decline in physical function are experienced by older women with greater physical function at baseline.

### Ethnicity/Geography

#### Muscle Mass

Data from the Boston Area Community Health and Bone Survey showed higher lean mass index in black (*p* < 0.001) and Hispanic (*p* = 0.06) men when compared with white men after adjustment for confounding influences [33]. Black subjects in the Health ABC study were also found to have higher appendicular skeletal muscle than whites [19]. Results from The Third National Health and Nutrition Examination Survey found similar ethnic differences with significantly higher FFM and FFMI found in black compared to white women. The difference was not found to be significant in men [34]. When compared with data from black and white populations from two US studies, Auyeung et al. [24] found ASM appeared to be lower in a Chinese population; however, after adjustment, height appeared to account for much of this difference. Goodpaster et al. [19] noted African American participants lost more leg lean mass than whites in both absolute and proportional terms over a 3-year period. However, Wu and colleagues noted very similar rates of muscle mass decline between Asian, black and white populations [35].

#### Muscle Strength

In the Health ABC Study muscle strength was lower in black compared to white men and women, despite the higher measures of lean mass observed in these groups [19]. Black participants experienced greater declines in muscle strength when compared to white participants (*p* = 0.001) [19]. When Asian populations are compared to other ethnic populations lower muscle strength has been described [24]. Auyeung et al. [24] observed a decline in grip strength in Chinese participants that was more rapid than that of ASM and gait speed. Over a 2-year period, women experienced a 10.0% decline, while men experienced a 3.85% decline. When compared to other ethnic populations, the rate of decline in muscle strength was much more rapid in Asian populations [35].

**Fig. 2:**
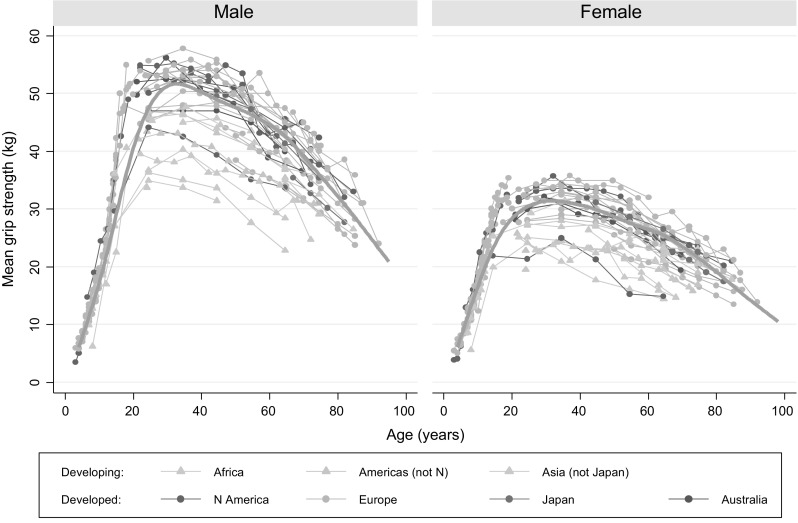
Grip strength mean values from included samples, by UN region. *Each point* represents the mean value of grip strength for each item of normative data, plotted against the mid-point of the age range it relates to. Values from the same sample are connected. Data from developing and developed regions are shown with *triangles* and *circles*, respectively. For comparison, the *grey curve* shows the mean values from our normative data for 12 British studies [36]

A systematic review and meta-analysis confirms marked global variations in grip strength [36]. A similar pattern to that observed in a combined British cohort [25], with increases in early life, maintenance of strength in mid-life and a decline in later life, was observed in this data synthesis. Mean grip strength in developing countries was substantially lower than that in developed countries [36], raising the possibility that region-specific reference values may be necessary for this widely used measure (Fig. 2).

#### Physical Function

As with the relationship observed between muscle mass and muscle strength in non-white men, it has been reported that higher muscle mass does not translate into better physical functioning in certain ethnic groups. Araujo et al. [33] found that the higher values of lean mass observed in black and Hispanic men did not correspond with better physical functioning in these subgroups when compared with white men. Significant differences in physical function have also been observed between ethnic groups in other studies. In the USA, non-Hispanic blacks were shown to have the lowest physical performance summary score when compared to Mexican American and non-Hispanic whites [37]. The relationship between ethnicity and physical performance was significantly influenced by socio-economic status, health and medical factors. Similar findings have been reported in other studies, African American women aged 45–79 years were 3 times more likely [OR 2.9 (95% CI 2.0, 4.1)] to have a gait speed of <1.0 m/s when compared to white Americans of the same age [38].

Auyeung et al. compared gait speed from a cohort of older Chinese adults to a systematic review of gait speed for adults aged 70–80 years. They observed slower gait speed among the Chinese cohort (1.07 and 0.96 m/s compared to 1.33 and 1.13 m/s for men and women respectively); however, statistical analyses were not carried out to identify if these differences were significant [24].

Rates of decline in physical functioning appear to follow a similar pattern as those described for muscle strength with the most rapid declines being experienced among Asian populations and the most gradual declines shown in white populations [24, 39].

### Secular Trends

Temporal trends have been studied for a number of musculoskeletal outcomes with prospective studies suggesting an increase in incidence of these conditions over recent decades. Part of the increased frequency of these disorders (e.g. osteoporosis, osteoarthritis and regional musculoskeletal pain) in the population is attributable to the global rise in life expectancy and resulting population ageing.

**Fig. 3:**
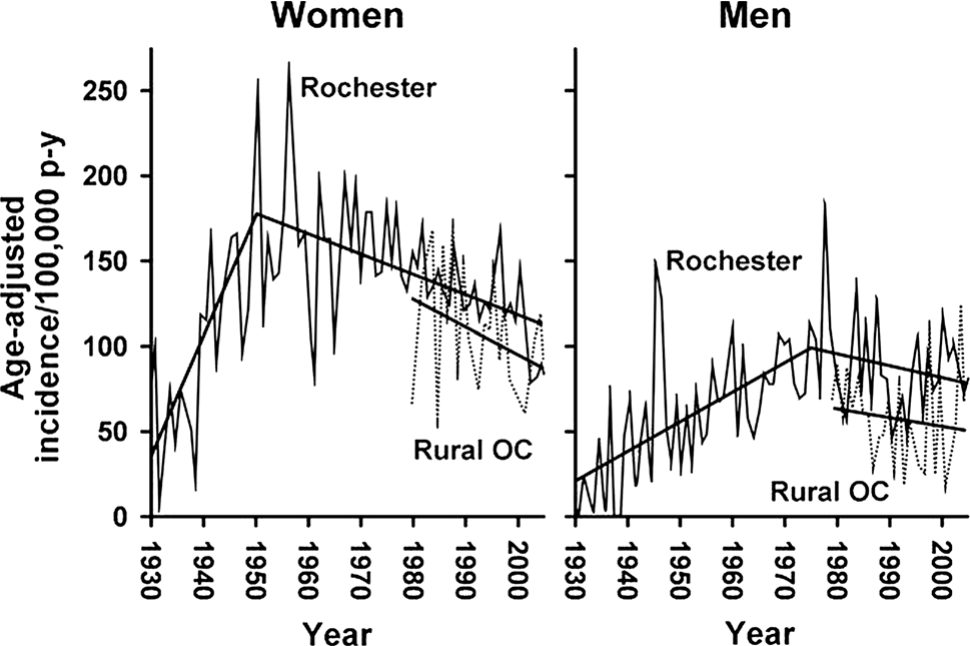
Age-adjusted incidence (per 100,000 person-years) of first-ever hip fracture among women and men residing in Rochester (1928–2006) or rural Olmsted County (1980–2006), Minnesota, by calendar year [41]

Data from a number of western populations have reported steep increases in the incidence of hip fracture over the last century [40]. The longest standing cohort data arise from the Rochester Epidemiology Project, where rates rose from 1928 until around the 1970s. In most other North American, European and Oceanic studies, rate rises continued until the 1990s. However, extension of the period of investigation until the present has suggested that rates in these countries have actually begun to plateau, and in some instances, even to fall (Fig. 3) [41]. Age–period–cohort models suggest contribution to this secular trend from both period and birth cohort effects, pointing at environmental influences during later life, as well as factors acting during development. As with the reported incidence in hip fracture, fall-related hospital admissions in the Netherlands rose between 1981 and 2008 [42]. The overall incidence rate increased by 61% with an annual age-adjusted incidence growth of 1.3% for men and 0.7% for women (*p* < 0.001 for the difference between the genders). Finally, data from the Fels Longitudinal Study [43] provide evidence of an increasing secular trend in peak grip strength with evidence for birth cohort and period effects. However, no cohort was observed over the entire adult lifecourse in this analysis, and the relationship between grip strength in early and late adulthood, as well as the tracking of rates of change in grip strength remains important research questions.

In summary, sarcopenia has been shown to be associated with an increased risk of falls in older adults [44]. The secular trends observed in hip fracture and fall-related hospital admissions are likely to reflect, at least in part, changes in muscle quality and physical functioning in successive generations of older adults but further research with measurement of key muscle-related outcomes is required. Appropriate data on the health impact and economic consequences of sarcopenia also need to be collected, to contextualise its position in the hierarchy of medical need in economically stressed healthcare systems. Finally, an understanding of environmental risk factors will generate an understanding of the true economic and health impact of the condition.

## Risk Factors

### Body Build and Obesity

#### Muscle Mass

Ageing is associated with major changes in body build and composition, notably decreased muscle mass, decreased height and increases in fat mass [45]. These changes can cause difficultly in interpreting older adult’s BMI as loss of height results in a higher BMI, or overestimation of fat mass [46]. A decrease in lean body mass is frequently offset by increases in fat mass, often presenting as a stable weight and BMI [46]. Newman and colleagues noted that when older adults lost and then regained weight they experienced an overall net loss of lean mass [47]. However, exercise and in particular progressive resistance training have been shown to attenuate the loss of muscle mass induced by weight loss in older adults [48].

Increased fat mass has been shown to be associated with greater muscle mass and also increased rates of decline in leg lean mass (0.02 kg per year, *p* < 0.01) in both men and women aged 70–79 years over an 8-year period [49]. The combination of obesity and low muscle mass has been termed as sarcopenic obesity and has been described in a number of other reviews [46, 50].

#### Muscle Strength

Data from the European Prospective Investigation into Cancer-Norfolk study [51] investigated obesity, as defined by BMI and waist circumference, in relation to grip strength. Higher BMI and WC were shown to be independently associated with increased grip strength in men but not women, with a stronger association existing for BMI. When BMI was considered in categories, men and women who were classed as underweight both had significantly lower grip strength than those with higher BMI. Considering both BMI and WC in the same model showed that WC was negatively associated with grip strength in both sexes suggesting a detrimental role of abdominal obesity on strength in older adults [51].

#### Physical Functioning

Participants in the Australian Longitudinal Study of Ageing with a high waist circumference [>102 cm (men) or >88 cm (women)] had increased odds, when compared with participants with lower waist circumference, of self-reported physical function limitations (OR 1.86, 95% CI 1.30, 2.65) after 2 years [52]. High waist circumference was also shown to be associated with poor physical function in very old (90 + years) women but not men of the same age [53].

Men and women in the HALCyon cohort demonstrated lower physical performance with increasing BMI [54]. This cross-sectional association appeared in a non-linear trend with the poorest measures of physical performance being observed in the most overweight group and suggestions of weaker performance in underweight groups. A similar, curve–linear relationship has been observed by Rejeski et al. [55]. These data make the suggestion of a threshold effect at which BMI, at its extremities, becomes detrimental for physical functions, a possibility that has previously been suggested in a review by Vincent et al. [56]. Evidence exists to suggest a sex difference in the relationship with BMI and physical functioning with a stronger association between increased BMI and decreased physical functioning being observed for women [54, 56].

### Physical Activity

#### Muscle Mass

Developments in the uses of accelerometer-defined physical activity (PA) levels have been beneficial as it allows for objective comparisons of different intensities of PA. Foong et al. [57] compared accelerometer-defined PA with muscle mass in community-dwelling older adults and noted significant positive associations with light [1.5–2.9 Metabolic equivalents (METS)], moderate (3–5.9 METS) and vigorous activity (≥ 6 METS) and percentage lean mass, with a dose–response effect indicating the largest effects for vigorous activity. Park et al. [58] found similar results in a Japanese cohort, aged 65–84 years, but only found significant associations with muscle mass for PA measures in the moderate and vigorous ranges.

Research has shown that the amount of time spent in sedentary behaviours is higher for older adults than other age groups and is associated with a morbidity and mortality [59]. Evidence suggests that older adults are at particular risk of rapid rates of muscle loss during periods of prolonged bed rest which are often induced by periods of ill health and hospitalisation [60]. Studies describing the relationship between sedentary lifestyles and muscle mass in healthy older adults tend to describe a negative influence of sedentary behaviours such as television watching and sitting [57, 61]. Foong et al. [57] showed that sedentary activity, defined as <1.5 METS, was associated with lower percentage lean mass in older adults *β* = − 0.1; 95% CI −0.1, −0.03; *p* < 0.001.

#### Muscle Strength

A systematic review and meta-analysis of 17 studies [62] documented a moderate effect of PA and muscle strength in adults aged 40–65 years (SMD 0.54, 95% CI 0.38, 0.70). Ferreira and colleagues [62] noted larger effect sizes in studies that included resistance exercises, use of weights with a moderate to high intensity, (10 studies, SMD 0.68; 95% CI 0.49, 0.87) when compared to the studies that did not include this type of exercise (17 studies, SMD 0.32; 95% CI 0.09, 0.55). Marques et al. [63] found similar results to suggest resistance exercise to be more beneficial than aerobic exercise over an 8-month period for improving muscle strength in older adults. The beneficial effects of resistance training on muscle strength have also been described in other systematic reviews which particularly emphasise the benefits of resistance exercise when performed at higher intensities [64, 65].

There have been mixed reports regarding the role of PA throughout the lifecourse and its influence on muscle strength in later life. Data from the National Survey of Health and Development indicate a positive cross-sectional relationship at 53 years with PA and muscle strength in men but did not show any significant benefits of PA earlier in mid-life [66]. Dodds et al. [67] used the same dataset, after additional data collections, to show a positive association with leisure time PA and muscle strength in later life and suggested a cumulative benefit of greater activity which limits the rate at which muscle strength declines. One study found that participants who became physically sedentary during a follow-up period of 22 years had a significantly greater rate of grip strength decline than in those who maintained physically active throughout the follow-up period [68].

#### Physical Function

A number of previous studies in mid-aged and older adults have shown that individuals with higher levels of PA have better physical functioning [66, 69–71]. Studies have investigated the effects of both aerobic and resistance trainings on physical function. Results from the 2009 Cochrane Review [64] of PRT in older adults suggest that PRT may not be as beneficial in improving physical function as it is muscle strength. The results showed a small but significant improvement in physical ability (33 trials, 2172 participants; SMD 0.14, 95% CI 0.05, 0.22).

Evidence from The InCHIANTI Study [72] showed that older adults who reported regular moderate-to-vigorous PA during mid-life were significantly more likely to perform better of physical performance tests than those with lower levels of PA in mid-life. Other studies have shown similar evidence to suggest that PA throughout the entire lifecourse plays a role in improving physical performance in later life [66, 73, 74].

The negative impact of sedentary behaviours on physical function has been described in numerous studies and this relationship has often been found to be independent of other PA [75–78]. Data from the Women’s Health Initiative, USA, found that in each PA category (mild, moderate, strenuous) declines in physical function were greatest in the women reporting the most time spent in sedentary behaviours [77]. Evidence suggests that reducing the time spent in sedentary behaviours may also be beneficial to reducing the rate of declines in physical function in later life. Longitudinal data for 1659 community-dwelling men and women from the Osteoarthritis Initiative showed a significant relationship between loss of physical function over a 2-year period and time spent in sedentary behaviours at baseline [78]. Similar to the previous results from the Women’s Health Initiative, these results were independent of time spent in moderate-to-vigorous activity [78].

### Diet Pattern

Lower food intake is associated with ageing and is often due to a combination of physiological, social and psychological factors. This decreased intake can make it difficult for older adults to meet the recommended intake for certain nutrients. The high correlation between different food items presents a major problem when considering one nutrient in isolation [79]. Research investigating diet quality and dietary patterns has been considered useful for gaining an insight into the influence of the diet as a whole and its influence on health.

A number of epidemiological studies have considered muscle outcomes in relation to dietary patterns. A “healthier” diet, as characterised by high consumption of fruit, vegetables, whole-grain cereals and fatty fish, has been shown to be associated with higher grip strength in community-dwelling older adults [80]. The most intensively studied dietary pattern is the Mediterranean Diet with associations being observed with increased Mediterranean Diet adherence and improved walking speed [81–83] and reduced risk of frailty [82, 84].

Most research investigating the influence of diet in sarcopenia has focused on individual nutrients rather than the diet as a whole. Some specific nutrients have been noted to be of particular interest in relation to sarcopenia.

### Protein

#### Muscle Mass

Insufficient protein intake has been linked to decreased muscle mass in a number of epidemiological studies. The Health ABC Study examined the relationship between protein intake and muscle mass in community-dwelling older men and women [85]. Over a 3-year period, a greater loss of lean mass, assessed using dual-energy X-ray absorptiometry, was observed in the participants in the lowest quintile of protein intake at baseline. This group lost 40% more lean body mass than those in the highest quintile at baseline [85].

A number of groups have suggested that the daily recommended protein intake of 0.8 g/kg body weight per day for adults may be inadequate as even a minimum value for older adults and recommended further research into optimal protein intakes for this older population [86–88]. Recommendations have been made for a protein intake of 1.0–1.2 g/kg body weight per day and are thought to be an optimal amount to maintain skeletal muscle health without affecting renal function in older adults [87, 88]. A study of community-dwelling older adults in Southern Tasmania, Australia, showed that failing to meet the Australian and New Zealand recommended dietary intake (RDI) for protein (64 and 81 g/day for men aged 51–70 years and >70 years, respectively, and 46 and 57 g/day for women in the same age groups) was associated with significantly lower ALM at baseline (−0.81 kg, 95% CI −1.54, −0.08; *p* = 0.03) and follow-up (−0.79 kg 95% CI −1.42, −0.17; *p* = 0.01) [89].

Intervention studies have shown mixed findings for the use of protein/amino acid supplementation in older people with a number of studies describing an association with greater muscle mass [90–92] and other trails showing no increases in muscle mass [93, 94]. A recent systematic review and meta-analysis of 22 trails demonstrated that protein supplementation during prolonged periods of resistance exercise showed positive effects for fat-free mass when compared with a placebo (Weighted Mean Difference (WMD): 0.69; 95% CI 0.47, 0.9; *p* < 0.01) [95].

#### Muscle Strength

Results from observational studies have been conflicting when describing the relationship between protein intakes and muscle strength. Positive associations of borderline significance were described between % energy from protein and grip strength in the Hertfordshire Cohort Study [80]. No significant association was found between protein intake and muscle strength in community-dwelling older adults in Southern Tasmania [89].

A 2009 Cochrane review [96] performed a meta-analysis of 7 studies, 593 participants, that had investigated the effect of protein supplementation on handgrip strength with results showing no demonstrable effect (WMD 0.06; 95% CI −0.60, 0.72). However, results from a 2012 meta-analysis showed protein supplementation combined with resistance training to have beneficial effects on 1-RM leg press strength [95].

#### Physical Function

A 2009 Cochrane review by Milne et al. [96] concluded that the evidence did not show any improvements on functional measurements with protein supplementation. More recent supplementation trails have found mixed results. A study in community-dwelling Australian women found no significant effects of whey protein supplementation on physical functioning measures over a 2-year period [97]. However, on the contrary, evidence from a small intervention study by Tieland et al. [92] observed that protein supplementation improved physical performance measurements in frail older adults. The results showed a significant increase in SPPB score (8.9 ± 0.6 to 10.0 ± 0.6 points) in those that received the protein supplementation [92].

### Vitamin D

#### Muscle Mass

The relationship between vitamin D and muscle mass remains uncertain [98]. Significant, positive associations have been observed between serum 25-hydroxyvitamin D [25(OH)D] and ALM in frail older adults; however, these associations appear to be rather modest (*β* = 0.012, *p* = 0.05) [99]. Similar results were observed in 686 community-dwelling older adults from Tasmania [100] with cross-sectional results showing a positive association between 25 (OH)D and ALM at both baseline and follow-up. Baseline 25(OH)D status however did not predict % ALM at follow-up (2.6 years) [100].

A recent systematic review of vitamin D supplementation trials found no significant association with muscle mass; however, only six studies were included in this meta-analysis and further research in this area is required [101].

#### Muscle Strength

A number of observational studies have described significant associations between hypovitaminosis D and lower muscle strength. Data from the Longitudinal Aging Study Amsterdam showed that participants with baseline 25-OHD levels below <25 nmol/l were at 2.57 (95% CI 1.40, 4.70) greater odds of suffering from sarcopenia, defined as a the lowest sex-specific 15th percentile of the cohort for grip strength [102]. A more recent study conducted among Argentinian women aged 65 + years found that women with 25-OHD levels ≥20 ng/ml had significantly stronger knee extensor and hip abductor muscles [103].

A recent systematic review and meta-analysis showed a small positive impact of vitamin D supplementation on muscle strength (SMD 0.17; 95% CI 0.03, 0.31; *p* = 0.02) [101]. When this meta-analysis investigated strength measurements separately, no significant association was found for grip strength (SMD 0.01; 95% CI −0.06, 0.07; *p* = 0.87) but a positive association was found for lower limb strength (SMD 0.19; 95% CI 0.05, 0.34; *p* = 0.01) [101].

#### Physical Function

Observational data have shown significant associations for declining vitamin D status in relation to deteriorating physical functioning in older adults [104]. Wicherts and colleagues observed that men and women with 25(OH)D <10 ng/ml and 25(OH)D between 10 and 20 ng/ml had significantly higher odds for decline in physical performance, when compared to participants with 25(OH)D of at least 30 ng/ml, over a 3-year period (OR 2.21; 95% CI 1.00, 4.87; and OR 2.01; 95% CI 1.06, 3.81) [105].

A systematic review of the effects of vitamin D supplementation on muscle strength, gait speed and balance in older adults, published in 2011, showed evidence that vitamin D supplementation had positive effects on physical functioning with improvements shown for postural sway and time to complete the Timed Up and Go Test in older adults [106].

### Micronutrients/Other

The anti-inflammatory properties of omega-3 (n-3) fatty acids have been suggested to be beneficial to muscle mass, strength and function. A small randomised control trial found that n-3 fatty acid supplementation for an 8-week period improved the hyperaminoacidemia–hyperinsulinemia-induced increase in the rate of muscle protein synthesis in older adults and suggested that n-3 fatty acids could be used as a potential therapeutic agent to address the age-related loss of muscle mass [107]. Data from the Hertfordshire Cohort Study have shown that grip strength is associated with fatty fish consumption in men and women with each additional portion of fatty fish consumed per week being associated with an increase of 0.43 kg (95% CI 0.13, 0.74; *p* = 0.005,) in men and 0.48 kg (95% CI 0.24, 0.72; *p* < 0.001) in women in grip strength [80].

Data from the InCHIANTI study have shown positive associations between plasma concentrations and dietary intake of antioxidants, in particular vitamin C and β-carotene and skeletal muscle mass [108] as well as the associations between higher total plasma carotenoids lower the risk of developing severe walking disability and a reduced rate of decline in 4-m walking speed over a 6-year follow-up [109].

### Smoking

#### Muscle Mass

A recent meta-analysis [110] concluded that smoking may have little impact on the development of sarcopenia, and results from studies remain inconclusive. The majority of studies included in this meta-analysis used muscle mass to define sarcopenia. Separate studies have considered the relationship between smoking and muscle mass as part of wider lifestyle analysis and found varied results. A cross-sectional study by Szulc et al. [111] found that men who were current smokers had lower relative appendicular skeletal muscle mass index than those who never smoked (−3.2%; *p* < 0.003). Similar results have been reported by Baumgartner et al. [112]. In contrast, other authors have reported that smoking is not an important risk factor for low muscle mass when considered in fully adjusted models [113].

#### Muscle Strength and Physical Function

Cross-sectional associations have been described between smoking and decreased muscle strength in older adults [114, 115]. A longitudinal study in healthy younger adults showed smoking to be inversely associated with knee muscle strength between the ages of 21–36 after adjustment for other lifestyle factors [116]. Data from HALCyon have shown a strong association between smoking and reduced physical capability as measured by grip strength, chair rise speed, TUG/walk speed and balance ability [114]. The strongest association was observed with current compared to never smoker status when considered in relation to walking and TUG speed [*Z* scores −0.23 and −0.29, respectively (*p* < 0.0001)].

### Alcohol

Few studies have investigated alcohol as a primary focus in relation to muscle mass, muscle strength and physical function in older adults but like smoking, alcohol has been considered in some studies as a part of wider lifestyle analyses. A recent systematic review and meta-analysis of these studies (*n* = 9) showed that alcohol consumption did not contribute to the development of sarcopenia [117]; however, a number of limitations were noted by the author including differences in methods used to measure alcohol consumption and the continuing problem of a lack of an agreed universal definition for sarcopenia.

### Co-Morbidity

The prevalence of sarcopenia has been shown to be higher in patients presenting another health condition [118]. However, little evidence exists to describe the risk of individual co-morbidities and muscle mass, strength and physical function separately.

The presence of many chronic illnesses such as chronic obstructive pulmonary disorder (COPD), cardiovascular disease and cancer have been shown to be associated with loss of muscle mass. The wasting of muscle in relation to chronic illness is referred to as cachexia [119, 120] and can occur at any age but is particularly common with increasing age. Prevalence of sarcopenia, defined by gender-specific lean body mass cut points, was found to be high in Chinese patients receiving treatment for cancer, with 96 out of 113 patients having the condition [121]. In this study, men with cancer were found to have a greater risk of developing sarcopenia than women [121].

Type 2 Diabetes has been shown to be associated with loss of muscle [122] as well as declines in muscle strength [122–124] and physical performance [125–127]. Park et al. [122] demonstrate that participants with diagnosed and undiagnosed type 2 diabetes experienced greater rates of decline in loss in muscle mass compared with the participants who did not have type 2 diabetes independent of weight loss over time.

The Sarcopenia and Translational Aging Research in Taiwan (START) study has shown that increasing numbers of co-morbidities are associated with lower grip strength and physical function measures, walking speed and TUG, in older adults [128]. These associations are strengthened in the presence of low muscle mass. Participants with two or more chronic diseases and low muscle mass performed more poorly than those with no risk factors after adjustment for confounding factors [128].

Other conditions such as coronary heart disease/congestive heart failure and vision problems have been shown to be significant predictors of lower muscle strength [123]. Even though associations between certain co-morbidities and muscle mass, strength and function have been shown in the literature, it is worth considering that these relationship may be mediated by a number of factors such as lower levels of physical activity and higher numbers of inflammatory markers [45].

### Combined Lifestyle Factors

Unhealthy lifestyle choices have been shown to coexist in individuals [129] and a few studies have reported the association of combined poor health behaviours and domains of sarcopenia. Robinson et al. [130] showed a strong inverse and graded association between number of poor lifestyle risk factors (smoking, obesity, poor diet and low physical activity) and physical functioning in men and women. After adjusting for confounders, a four times greater risk of poor self-reported physical function was reported in men who had three or four lifestyle risk factors (vs none) and a five times greater risk in women.

**Fig. 4:**
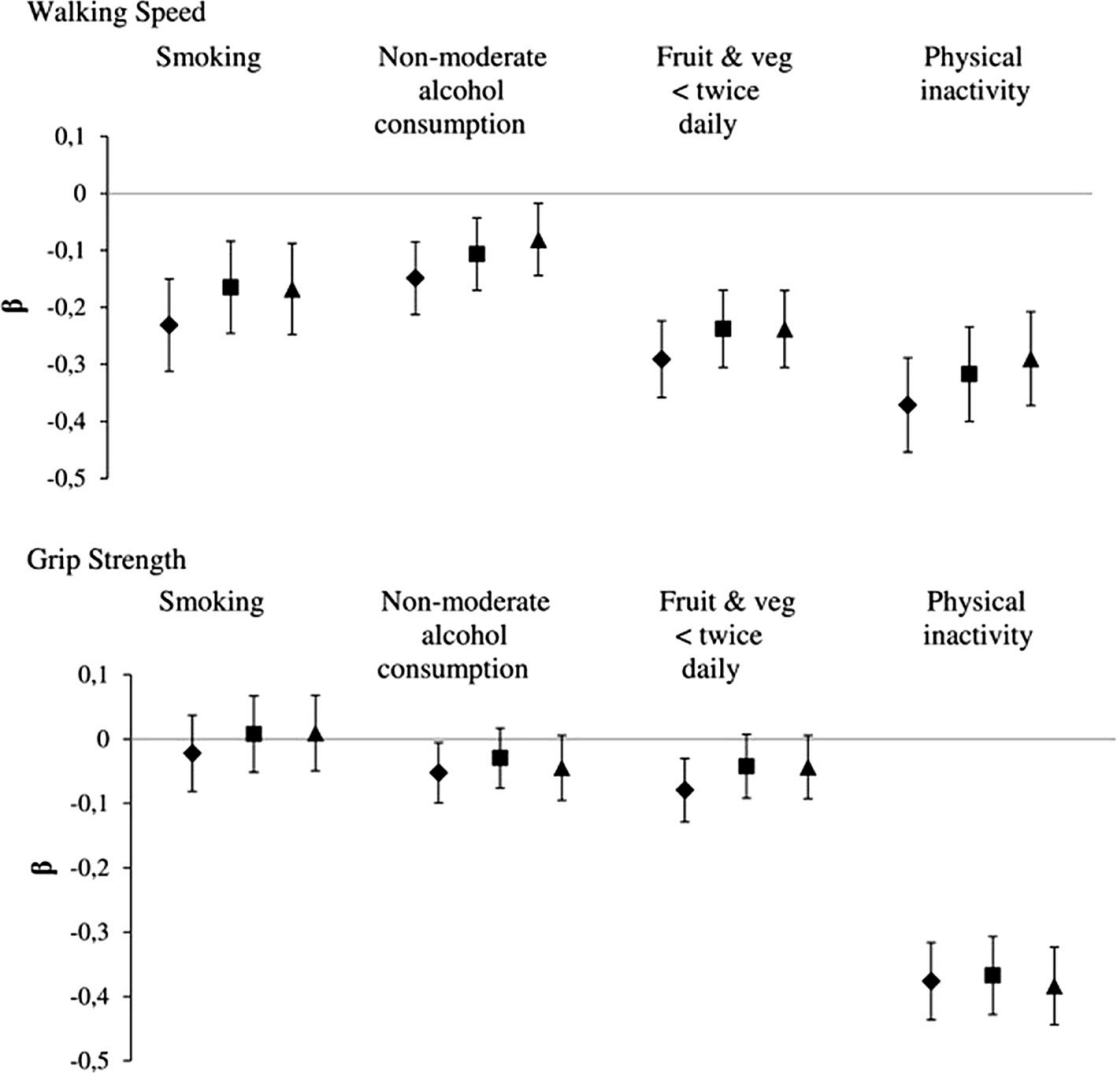
Cumulative effect of unhealthy behaviours (1991–1993 to 2002–2004) on physical functioning in 2007–2009 before and after mutual adjustment for health behaviours, and additionally adjusted for body mass index (BMI). *β* represents mean difference in standardised score of physical functioning. Models are adjusted for age, sex, educational level, marital status and height (and mutually adjusted for health behaviour scores for *bold square* results). Estimates are for a 1-point increment in cumulative score of the unhealthy behaviour under consideration assuming a linear association between the number of times a person was classified as having the unhealthy behaviour in the three assessments (1991–1993, 1997–1999 and 2002–2004) and physical functioning. *Filled diamond* each health behaviour separately, *filled square* health behaviours mutually adjusted, *filled triangle* additionally adjusted for BMI [131]

Similarly, the cumulative association between adult health behaviours assessed 5, 10 and 17 years before measures relating to sarcopenia has been shown in data from the Whitehall II study [131]. Results showed that all mid-life measured unhealthy behaviours (smoking, non-moderate alcohol intake, low fruit and vegetable consumption and physical inactivity) were associated with lower walking speed 17 years later. Figure 4 shows the cumulative effect of unhealthy behaviours, measured between 1991–1993 and 2002–2004, on grip strength and walking speed in 2007–2009 before and after mutual adjustment. An association was found with cumulative scores for all 4 unhealthy behaviours in relation to walking speed independently and after mutual adjustment; however, only low fruit and vegetable consumption and physical inactivity showed clear evidence of accumulation-of-risk for walking speed. Only physical inactivity showed an accumulation of risk for grip strength after mutual adjustment [131].

These studies suggest that the coexistence and duration of unhealthy behaviours, in particular diet and physical inactivity, may have a profound effect on sarcopenia risk, particularly physical functioning. Efforts to encourage healthy lifestyle choices throughout life have the potential to improve physical function at older ages.

### Developmental Programming

The term developmental programming is used to describe the influence of exposures that occur during critical developmental periods in early life and the subsequent lasting effects on various systems in the body [132]. Epidemiological studies into the Developmental Origins of Health and Disease (DOHaD) have shown associations between low birth weight and weight a one year, markers of poor intrauterine and early life and a range of health conditions in later life including cardiovascular disease, osteoporosis and sarcopenia [133–136].

#### Muscle Mass

In the Hertfordshire Cohort Study (HCS), birth weight and weight at 1 year were strongly correlated with fat-free mass in 737 older community—dwelling men and women [137]. Similarly, a cross-sectional study in Helsinki found a 1 kg increase in weight at birth which corresponded to a 4.1 kg (95% CI 3.1, 5.1) increase in adult lean mass in men and a 2.9 kg (95% CI 2.1, 3.6) increase in women [138].

#### Muscle Strength

**Fig. 5:**
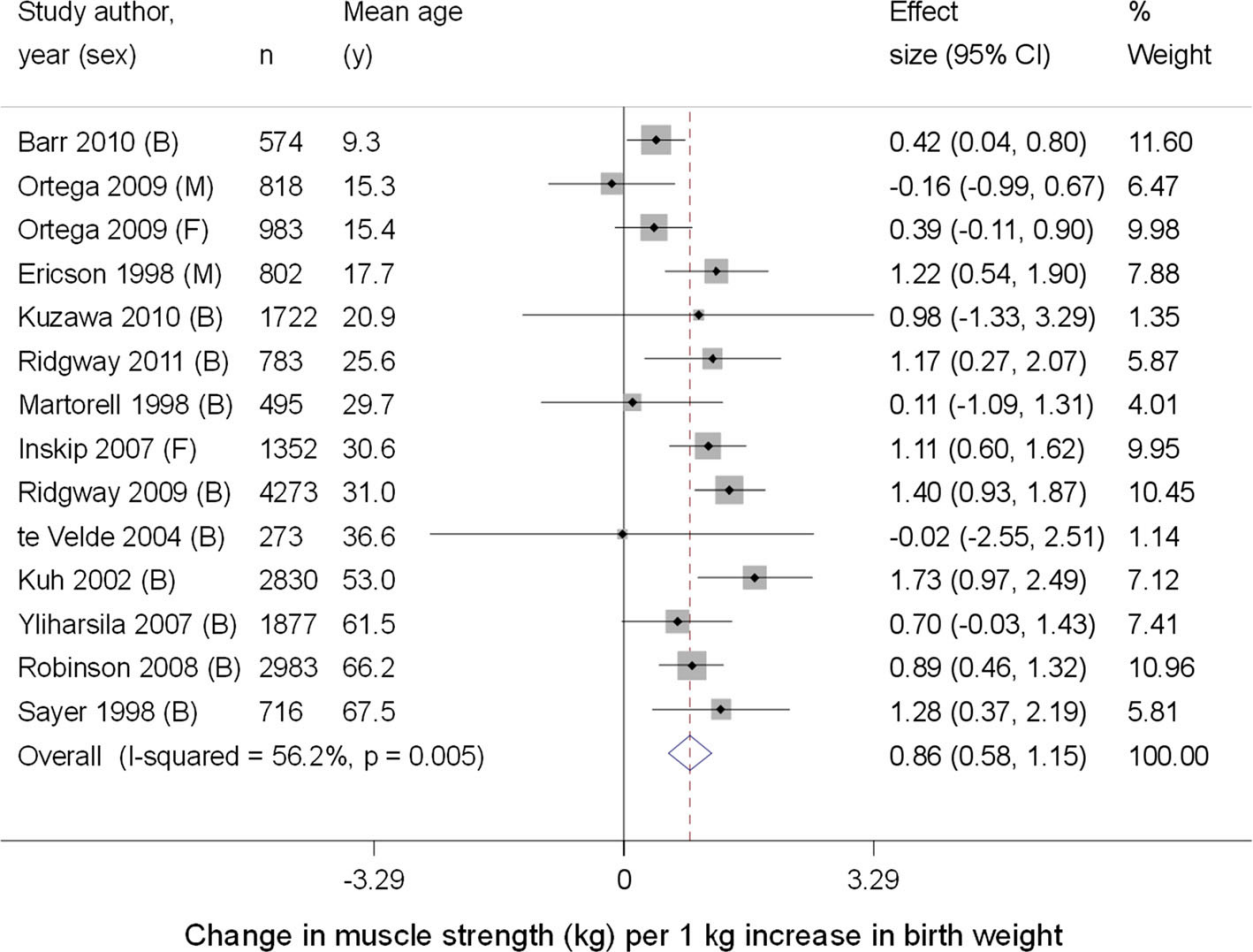
Forest plot of studies assessing the association between birth weight (kg) and later muscle strength (kg), after adjustment for age and height. Studies ordered by mean age at time of strength measurement. *B* both males and females, *M* males only, *F* females only included in study [139]

A 2012 systematic review and meta-analysis found that 17 studies showed a positive association between higher birth weight and increased muscle strength. The meta-analysis included 13 studies, 20,481 participants, and showed a 0.86 kg (95% CI 0.58, 1.15) increase in muscle strength per additional kilogram of birth weight, after adjustment for age, gender and height at the time of strength measurement (Fig. 5) [139]. Similar associations have been observed with increased weight at 1 year being associated with increased grip strength in adult life [140]. Early life feeding has also been shown as having a potential influence on muscle strength in later life. In the Hertfordshire Cohort Study, longer duration of breastfeeding was associated with higher grip strength in older men (mean age 66 years) [141].

#### Physical Function

The relationship between birth weight and physical functioning in later life has not been as widely researched. Evidence from Von Bonsdorff et al. [142] reports a lower SF-36 physical functioning score in older adults who had a birth weight of 2.5 kg or lower when compared to those weighing 3.0–3.5 kg at birth (OR 2.73, 95% CI 1.57, 4.72). Lower birth weight was shown to be associated with poor balance in men in the Hertfordshire Cohort Study, but not with other measures of physical functioning [143]. This study concluded that adult lifestyle factors may be more influential in determining physical functioning in older adults than development factors.

## Conclusion and Future Direction

This review of the epidemiology of sarcopenia has documented evidence of the differential peak and rate of decline for three components linked to the disorder: muscle mass, strength and physical function. Differences are also apparent in relation to the peak level and subsequent loss rate of these characteristics between men and women; between ethnic groups and over time. The data suggest that the rate of decline in muscle mass is much less rapid than that in muscle strength. This, in turn, is much less pronounced than the rate of decline in physical function. Men have significantly higher levels of muscle mass, strength and function at any given age, than women. In contrast, rates of decline seem similar between the genders, for each of the three characteristics.

Ethnic differences are apparent in muscle mass, strength and function. Black populations have been noted to have higher levels of muscle mass, than white and Asian populations. The higher levels of muscle mass that are observed in some ethnicities do not translate into higher levels of muscle strength and function. Non-white populations are reported as experiencing a more rapid decline in muscle strength and functioning. Asian populations tend to have similar declines in muscle mass to non-Asian but experience much more rapid deterioration in strength and functioning.

Temporal trends have been much less studied for sarcopenia, than for osteoporosis and hip fracture. It is now clear that age and sex-specific incidents rate for hip fracture showed increases through the latter half of the last century, followed by a plateau and the beginning of a decline in recent years. This secular trend has been replicated in North America, Europe and Oceania. It is contributed to by both period and birth cohort affects. Similar age–period–cohort models are required for measures of muscle mass and strength; limited evidence suggests important components during development as well as involution.

Environmental risk factors for all three components of sarcopenia include sedentary lifestyles, adiposity and multi morbidity. The role of cigarette smoking and alcohol consumption are much less apparent than have been observed in studies of osteoporosis or cardiovascular disease.

Nutrition has been identified as having an important influence on the development of sarcopenia; in particular, protein intake has the potential to slow the loss of muscle mass, but does not appear to be as influential as in maintaining muscle strength or physical function. Physical activity, in particular resistance training, when performed at higher intensities appears beneficial for muscle strength and functioning. Trials combining protein supplementation and physical activity show promising results in reducing the decline in muscle strength and function with advancing age.

These descriptive characteristics of muscle mass, strength and function point to the urgent need for a consensual definition of sarcopenia incorporating these parameters. The FNIH Sarcopenia project [144] is pooling data from large well-characterised cohorts in an effort to identify clinically relevant thresholds for muscle mass and strength that may be generalised to both genders; different ethnicities; multiple geographic regions; as well as a range of health states. The completion of this work will permit evaluation of novel preventive and therapeutic strategies in both individuals and larger populations.
